# Rupture of Umbilical Hernia with Evisceration in a Newborn - A Case Report

**DOI:** 10.21699/jns.v6i3.565

**Published:** 2017-08-10

**Authors:** Dinesh H Kittur, Kailas P Bhandarkar, Santosh V Patil, Sudhakar S Jadhav

**Affiliations:** Department of Pediatric Surgery, Sushrut Jadhav Kinderchirurgie Charitable Trust’s Paediatric Surgery Centre and PG Institute, Sangli, Maharashtra, India

**Keywords:** Umbilical hernia, Incarceration; Evisceration, Umbilical defect

## Abstract

Most umbilical hernias in infants do not need surgery and the ring will eventually close. Occasionally few complications can arise and incarceration is most common. Spontaneous rupture of the hernia and eventual evisceration is a rarely seen complication. A 3-week-old neonate having umbilical hernia presented with rupture of the sac with evisceration of bowel within a few days of first visit. No underlying cause like umbilical sepsis was found. The baby had emergency repair of the hernia with an uneventful recovery.

## INTRODUCTION

Most umbilical hernias present in the newborn period or early infancy. They rarely require surgical treatment [1,2] . Even in an infant with a large hernia sac, the parents can be assured that most of these hernias will eventually close as the infant turns prone, stands up and starts walking. However, in cases of large defect, shiny skin or skin ulceration, the infant needs to be observed more closely as chances of spontaneous rupture and evisceration are likely. Herein we report a neonate with umbilical hernia who developed rupture of hernia and evisceration of bowel.

## CASE REPORT

A 3-week-old neonate (2.8kg) delivered by LSCS for prolonged labor was seen in the out-patient department by a pediatrician for an umbilical hernia developed within a few days after the cord fell off. There was no history of any predisposing factors like umbilical sepsis, respiratory distress or abdominal distension. The umbilical sac measured 7x5cms, the skin was thin and shiny, with a small ulcer on the top. The umbilical defect was 2cms in diameter. The contents of the sac were easily reducible but appeared again. The parents were counseled about the possibility of the need for early surgery in view of the clinical findings. Three days later, the baby was admitted with a ruptured umbilical hernia and evisceration of the bowel (Fig.1). The neonate was otherwise normal. At surgery, the gut was returned to the abdomen and umbilical defect was repaired. The baby had an uneventful recovery and was discharged on the 4th day of surgery. The wound healed well.

**Figure:**
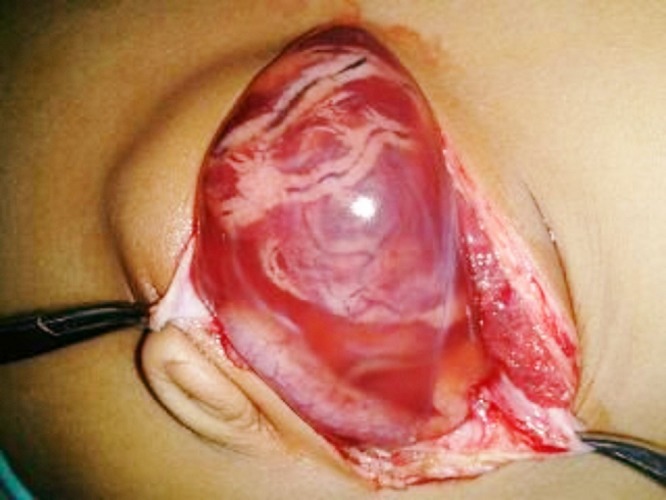
Figure 1: Ruptured umbilical hernia.

## DISCUSSION

In a newborn infant, the rectus abdominis muscles are wide apart. The linea alba is in the form of a band of about 20 mms width. The process of closure of the defect is hastened by the recti coming closer when the infant stands erect. Spontaneous rupture is a rarity and needs emergency closure of the defect [3]. Precipitating causes for spontaneous rupture and evisceration include umbilical sepsis, abdominal distension, respiratory distress [3], ventilatory support [2], ascites [4] and rarely Hurlers syndrome [5]. Early surgery may be indicated if warning signs of rupture are evident. If a child has a small umbilical defect with a large sac, the parents need to be counseled about the possibility of incarceration and subsequent need for early surgical repair. If a patient has a large defect and skin is stretched and shiny like in our case, increased intraabdominal pressure during crying is directly transmitted to the skin leading to ischemia and ulceration of the overlying skin. This mechanism is easily explained by Laplace law [6]. In the case of a small defect, the brunt of the pressure is partly borne by the linea alba, so the chances of rupture are small. In the case of large defect, like in our case, the tension is borne almost wholly by the skin. This can lead to ischemia of the skin, thereby predisposing to spontaneous rupture and evisceration.

To conclude, the treating physicians should be able to identify signs of skin ischemia like thin, stretched and shiny skin. In this situation, the child should be referred for surgical evaluation regarding early intervention.

## Footnotes

**Source of Support:** None

**Conflict of Interest:** None
